# Study on Horizon Scanning by Citation Network Analysis and Text Mining: A Focus on Drug Development Related to T Cell Immune Response

**DOI:** 10.1007/s43441-021-00351-3

**Published:** 2021-11-22

**Authors:** Erika Fujii, Takuya Takata, Hiroko Yamano, Masashi Honma, Masafumi Shimokawa, Hajime Sasaki, Mayumi Shikano

**Affiliations:** 1 Faculty of Pharmaceutical Sciences, Tokyo University of Science, 1–3 Kagurazaka, Shinjuku-ku, 162-8601 Japan; 2 Institute for Future Initiatives, The University of Tokyo, Bunkyo-ku, Japan; 3 Department of Pharmacy, The University of Tokyo Hospital, Bunkyo-ku, Japan; 4 Faculty of Pharmaceutical Sciences, Sanyo-Onoda City University, Sanyoonoda-shi, Japan

**Keywords:** Horizon scanning, Citation network, Text mining, Drug development, Immune, T cell, Immune checkpoint inhibitor

## Abstract

Certain innovative technologies applied to medical product development require novel evaluation approaches and/or regulations. Horizon scanning for such technologies will help regulators prepare, allowing earlier access to the product for patients and an improved benefit/risk ratio. This study investigates whether citation network analysis and text mining of scientific papers could be a tool for horizon scanning in the field of immunology, which has developed over a long period, and attempts to grasp the latest research trends. As the result of the analysis, the academic landscape of the immunology field was identified by classifying 90,450 papers (obtained from PubMED) containing the keyword “immune* and t lymph*” into 38 clusters. The clustering was indicative of the research landscape of the immunology field. To confirm this, immune checkpoint inhibitors were used as a retrospective test topic of therapeutics with new mechanisms of action. Retrospective clustering around immune checkpoint inhibitors was found, supporting this approach. The analysis of the research trends over the last 3 to 5 years in this field revealed several candidate topics, including ARID1A gene mutation, CD300e, and tissue resident memory T cells, which shows notable progress and should be monitored for future possible product development. Our results have demonstrated the possibility that citation network analysis and text mining of scientific papers can be a useful objective tool for horizon scanning of life science fields such as immunology.

**Supplementary Information:**

The online version contains supplementary material available at 10.1007/s43441-021-00351-3.

## Introduction

The application of innovative technologies that would lead to a product of novel modality or mechanism of action is expected to be a potential new therapeutic or diagnostic tool for diseases. However, there may be cases where the application of conventional development and evaluation concepts, regulatory frameworks, or both to innovative technologies are inappropriate. Therefore, early identification of innovative technologies with potential applications to medical products through horizon scanning would encourage regulatory authorities to establish new approaches to assess their quality, efficacy, and safety to advise developers and revise their regulations as needed. This could also contribute to timely patient access and improve the benefit-to-risk ratio of the product [1].

Traditionally, horizon scanning has been predominantly conducted in Europe for policy-making, scientific research funding, and healthcare budgeting purposes, by surveying the Internet, government, international organizations and companies, databases, and journals using the Delphi method [2, 3]. The IHSI, a regulatory entity with participation from eight European countries developed the IHSI Joint Horizon Scanning Database to promote fair and transparent pharmaceutical prices to drive price reduction, mitigate the impact of disruptive innovation, support effective budgetary policy and support HTA and regulatory preparation [4]. One of its recent activity is IHSI's agreement with Emergency Care Research Institute (ECRI), an independent healthcare service organization that provides technical solutions and evidence-based guidance to healthcare decision makers worldwide, to build the International Horizon Scanning Database [5]. The agreement will enable suppliers including government to develop a database of upcoming drug launches and IHSI high-impact reports, which may help level the playing field for stakeholders. In the field of medical product regulation, the International Coalition of Medicines Regulatory Authorities (ICMRA), a group of regulatory authorities from 30 countries and regions, recognized the need to respond quickly to innovative technologies and agreed on the importance of 'horizon-scanning' to identify such technologies [6].

Hines et al. reported that in the medical and healthcare field, most horizon scanning methods used were manual or semi-automated, with relatively few automated aspects. It is difficult to understand the whole picture of the extremely large and fragmented results of research and technological development. It might also be inappropriate to narrow the scope of consideration based solely on experts’ opinions, since information from experts might be subjective and the outcome depends on the choice of the expert. To solve this challenge, a computer-based approach can be used to complement the expert-based approach, as it fits the scale of the information [7, 8]. In particular, the citation-based approach assumes that the papers on which a paper is based and the papers it cites are similar. Analyzing this citation network allows us to understand the structure of the research areas constituting the large volume of papers that we can read. These methods have been widely used as powerful tools for visualizing and understanding the structure of a research field to identify new trends and research directions; they have been proven effective in various studies [9–11]. It has been reported that a citation network analysis can effectively and efficiently track emerging research areas in the field of sustainable science [12], including energy research [13], regenerative medicine [14], robotics [15], and gerontology [15]. Sakata et al. [16] proposed a meta-structure of academic knowledge on patent and innovation research to effectively assist policy discussions on intellectual property system reform. They has shown that network analysis and machine learning methods are useful for understanding and predicting the development of technologies such as solar cells [17] and nanocarbons [18] suggesting their approach is useful tools for R&D strategists and policymakers in various fields to understand the broad scope of scientific and technological research and make decisions for worthwhile investments in promising technologies.

In this study, we focused on T cell immunity, because the research and the market has grown rapidly to be one of the major fields for developing pharmaceuticals, but the research history of the field shows an unexpected complexity which is considered to be a feature significantly different from other scientific fields such as artificial intelligence and nanocarbon. We explored if Sakata’s method is able to apply to immunology field focusing on immune checkpoint inhibitors as a retrospective example with new mechanisms of action and identified new topics in this field optimizing the horizon scanning method according to the target field.

## Methods

### Extraction of Paper Data for Analysis

To select queries for citation network analysis and extract key articles to track the R&D history of immune checkpoint inhibitors, we selected the six key articles [19–24] shown in Table 1, which are milestones in the research history of immune checkpoint inhibitors, based on relevant reviews [25, 26] and the descriptions on the official page for the Nobel Prize in Physiology or Medicine 2018 awarded to Dr. James P. Allison and Dr. Tasuku Honjo [27].

**Table 1:** Key articles and the clusters in which they are contained

Label	Paper title	Published year	Cluster #	Times cited within each cluster
A	Induced expression of PD-1, a novel member of the immunoglobulin gene superfamily, upon programmed cell death	1992	5	95
B	CD28 and CTLA-4 have opposing effects on the response of T cells to stimulation	1995	Not found	Not found
C	Enhancement of antitumor immunity by CTLA-4	1996	2	222
D	Development of lupus-like autoimmune diseases by disruption of the PD-1 gene encoding an ITIM motif-carrying immunoreceptor. Immunity	1999	5	122
E	Engagement of the PD-1 immunoinhibitory receptor by a novel B7 family member leads to negative regulation of lymphocyte activation	2000	5	161
F	Involvement of PD-L1 on tumor cells in the escape from host immune system and tumor immunotherapy by PD-L1 blockade	2002	2	126

The key articles considered to be important milestones in the history of research and development of immune checkpoint inhibitors as well as the clusters obtained by citation network analysis of papers obtained from PubMed (see Table 2). The key article B was not included in the clusters formed

The keywords appearing in the key articles were used as search queries for paper titles, abstracts, author keywords, and keywords to extract papers, which included citation information for citation network analysis in the Web of Science literature database Web of Science Core Correction (WoS, Thomson Reuters) and PubMed (MEDLINE [Medical Literature Analysis and Retrieval System Online]). To avoid subjectively narrowing down the papers for analysis and to include as many key articles as possible, we used “immune*” AND “t lymph*” as queries for both PubMED and Web of Science. We obtained 132,433 papers from the PubMed search, of which 90,450 papers (68.3%) formed a citation network containing five of six key articles. From the WoS search, 41,880 papers were obtained, of which 37,297 papers (89.1%) formed a citation network, but only one key article was included. We used only papers obtained from PubMed, which are considered to cover more papers than WoS on the targeted immune checkpoint inhibitors for the following analysis of citation networks and text mining.

## Citation Network Analysis

**Fig. 1:**
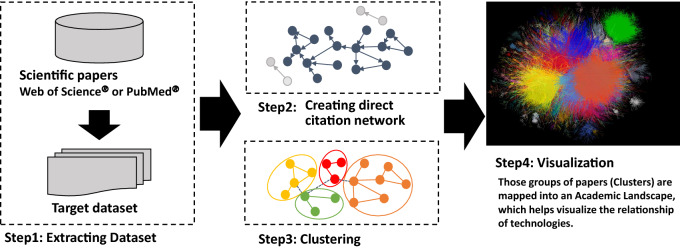
Steps of clustering and making academic landscape based on citation network [29, 30]. This Figure has been published in reference [18]. The procedure of the citation network is as follows: extraction dataset of academic papers for analysis (Step1). For the extracted dataset, the citation network was converted into an unweighted network with papers as nodes and citation relationships as links (Step 2). The network was then divided into several clusters using the topological clustering method (Step 3). In addition, a large graph layout (LGL) that is based on a force-direct layout algorithm displayed the largest connected component of the network to generate coordinates for the nodes in two dimensions, visualizing the citation network by expressing inter-cluster links with the same color (Step 4)

In this study, a citation network was converted into an unweighted network with papers as nodes and citation relationships as links. Papers with no citations as the largest component were considered digressional and were ignored in this study (Step 2 in Fig. 1). The core paper with the highest number of citations is located at the center of the citation relations. The network is then divided into several clusters using the topological clustering method. Topological clustering is a clustering method based on the graph structure of a network, and modularity maximization is used in the present scenario. Here, a cluster is a module in a citation network and a group of papers in which the citation relations are divided using a modularity (*Q* value) maximization method and are densely aggregated (Louvain method) [18, 28]. The modularity maximization method appreciates network partitioning such that the intracluster is dense and the intercluster is sparse. The modularity maximization method determines an optimal partitioning pattern by extracting the partitioning pattern that maximizes the modularity using a greedy algorithm. *Q* is an evaluation function of the degree of coupling within a cluster and between clusters, and is given as follows:

$$Q=\frac{1}{2m}\sum_{i,j}({A}_{ij}-\frac{{k}_{i}{k}_{j}}{2m})\delta ({c}_{i},{c}_{j})$$

where ${A}_{ij}$ represents the weight of the edge between $i$ and $j$, ${k}_{i}={\sum }_{j}{A}_{ij}$ is the sum of the weights of the edges attached to the vertex i, ci is the community to which vertex i is assigned, *δ*-function *δ*(*u*, *v*) is 1 if $u=v$ and 0 otherwise, and $m=1/2{\sum }_{ij}{A}_{ij}$.

The cluster No. labels are assigned numbers based on the number of papers included in the cluster. The characteristics of each cluster were confirmed by extracting a summary/abstract of frequently cited academic papers in the cluster and the characteristic keywords in the cluster.

In addition, we computed the term frequency-inverse cluster frequency (TF-ICF) to extract the characteristic keywords that were mechanically extracted by text mining with high TFCIF (Table 2) of each cluster. TF provides a measure of the importance of a term in a particular sentence. The inverse cluster frequency (ICF) provides a measure of the general importance of a term. The TF-ICF of a given term i in a given cluster j is given by

**Table 2:** Summary of clusters obtained from citation network analysis of papers obtained from PubMed

Cluster #	Median publication year	The number of papers	Top keywords	The title of the hub paper
1	2012	12,927	treg, regulatory, tregs, mouse, foxp3, cd4, autoimmune, tolerance	Control of regulatory T cell development by the transcription factor Foxp3
2	2012	12,911	tumor, cancer, immunotherapy, therapy, melanoma, vaccine, peptide, patient	Improved survival with ipilimumab in patients with metastatic melanoma
3	2007	8452	hiv, virus, siv, infection, vaccine, viral, infected, cd4	Temporal association of cellular immune responses with the initial control of viremia in primary human immunodeficiency virus type 1 syndrome
4	1984	7842	mouse, suppressor, antigen, tumor, suppressor cell, specific, induced, virus	Psychoneuroimmunology of psychological stress and atopic dermatitis: pathophysiologic and therapeutic updates
5	2009	7098	virus, hcv, infection, hepatitis, mouse, hbv, viral, influenza	Restoring function in exhausted CD8 T cells during chronic viral infection
6	2008	5859	dendritic, dendritic cell, tumor, vaccine, antigen, mouse, peptide, presentation	Dendritic cells and the control of immunity
7	1997	5804	mouse, tolerance, antigen, peptide, tumor, gamma, induced, specific	T cell tolerance by clonal elimination in the thymus
8	2008	4901	cmv, ebv, virus, infection, transplantation, hcmv, vzv, cytomegalovirus	Broadly targeted human cytomegalovirusspecific CD4 + and CD8 + T cells dominate the memory compartments of exposed subjects
9	2008	4019	tuberculosis, gamma, bcg, mycobacterium, infection, mtb, gammadelta, delta	Immunology of tuberculosis
10	2010	3289	tfh, mouse, tcr, activation, migration, synapse, tfh cell, antigen	Two-photon imaging of lymphocyte motility and antigen response in intact lymph node
11	2006	2800	parasite, malaria, vaccine, cruzi, infection, mouse, plasmodium, prrsv, leishmania	Gamma interferon, CD8 + T cells and antibodies required for immunity to malaria sporozoites
12	2011	2191	msc, ido, pregnancy, Mesenchymal, maternal, stem, mesenchymal stem, stem cell	Vitamin D antagonises the suppressive effect of inflammatory cytokines on CTLA-4 expression and regulatory function
13	2007	2050	tmev, abeta, cns, mouse, brain, dopamine, theiler, vip	Infiltration of CD4 + lymphocytes into the brain contributes to neurodegeneration in a mouse model of Parkinson disease
14	2008	1363	inkt, nkt, inkt cell, nkt cell, galcer, cd1d, alpha galcer, invariant	The role of NKT cells in tumor immunity
15	2013	1222	dengue, denv, sars, cov, covid, sars cov, zikv, wnv	Studies on production of biologically active substance which inhibits the intracellular multiplication of Toxoplasma within mouse macrophages
16	2010	1205	trachomatis, chlamydia, chlamydial, skin, chlamydia trachomatis, memory, hsv, infection	Memory T cells in nonlymphoid tissue that provide enhanced local immunity during infection with herpes simplex virus
17	2011	1177	atherosclerosis, hypertension, apoe, atherosclerotic, mouse, plaque, hypertensive, dahl	Role of the T cell in the genesis of angiotensin II induced hypertension and vascular dysfunction
18	2008	1083	diabetes, t1d, peptide, mouse, insulin, celiac disease, gluten, nod mouse, epitope	Translational mini-review series on type 1 diabetes: Systematic analysis of T cell epitopes in autoimmune diabetes
19	2016	681	tumor, covid, cancer, zebrafish, rainbow, mouse, igan, fish	Uncoupling the proinflammatory from the immunosuppressive properties of tumor necrosis factor (TNF) at the p55 TNF receptor level: implications for immune demyelination
20	2006	652	acaid, corneal, eye, anterior chamber, corneal allograft, dry eye, anterior, intraocular tumor	Comparative analysis of B and T cell epitopes of Mycobacterium leprae and Mycobacterium tuberculosis culture filtrate protein 10
21	2005	598	copd, tcdd, uranium, dolphin, htnv, pfos, chronic obstructive, obstructive pulmonary,	T cell-mediated hepatitis in mice infected with lymphocytic choriomeningitis virus. Liver cell destruction by H-2 class Irestricted virus-specific cytotoxic T cells as a physiological correlate of the 51Crrelease assay?
22	2004	571	htlv, ham/tsp, tax, atl, retinitis, virus, hbz, spastic paraparesis	Circulating CD8 + cytotoxic T lymphocytes specific for HTLV-I pX in patients with HTLV-I associated neurological disease
23	2005	456	pylorus, helicobacter, tick, helicobacter pylorus, pylorus infection, gastric, vaca, opn, microgravity	Sex hormones, immune responses, and autoimmune diseases. Mechanisms of sex hormone action
24	2003	384	rsv, rickettsia, tsutsugamushi, respiratory syncytial, syncytial virus, respiratory syncytial virus, rsv infection, virus	Role of T lymphocyte subsets in the pathogenesis of primary infection and rechallenge with respiratory syncytial virus in mice
25	2005	372	aav, vector, gene, hf.ix, transgene, gene transfer, dystrophin, gp19k	Induction of immune tolerance to coagulation factor IX antigen by in vivo hepatic gene transfer
26	2007	256	tirc7, pnh, cd26, aplastic, aplastic anemia, anemia, dpp4, peptidase	Cut to the chase: a review of CD26/dipeptidyl peptidase-4's (DPP4) entanglement in the immune system
27	2009	207	galectin, gal, sectm1, galectins, gal1, jet fuel, tungstate, galactoside binding	Targeted inhibition of galectin-1 gene expression in tumor cells results in heightened T cell-mediated rejection; A potential mechanism of tumor-immune privilege
28	1992	25	gangliosides, bbc, amalgam, glycophorin, bbc patient, pmps, cimetidine, amalgam restoration	Cimetidine as an immune response modifier
29	1998.5	12	p43, c48, fbl, c24d, plif, breast disease, plf, associated p43	Antibodies to placental immunoregulatory ferritin with transfer of polyclonal lymphocytes arrest MCF-7 human breast cancer growth in a nude mouse model
30	2017	10	butzleri, arcobacter, arcobacter butzleri, jejuni, butzleri infection, colonization resistance, jejuni-infected, butzleri induced	Survey of extra-intestinal immune responses in asymptomatic long-term Campylobacter jejuni-infected mice
31	1997.5	6	vita, iu/kg, vitamin, vita diet, dietary vitamin, reproductive performance, broiler breeder,chick	Effect of dietary vitamin A on reproductive performance and immune response of broiler breeders
32	2011	5	abrin, ricin temperature, pulchellin, ricin, temperature response, response gel, ricin temperature response, temperature response gel	Immunological response in mice bearing LM3 breast tumor undergoing pulchellin treatment
33	1975.5	4	psoriatics, treated with methotrexate, methotrexate, aliquots, lesion and lymphocyte transformation, skin lesion and lymphocyte transformation, psoriatics Treated, methotrexate had	Clinical aspects of T and B lymphocytes in psoriasis
34	2009	4	trem, sit and trim, linker for activation, transmembrane adaptor, lab, ntal, transmembrane adaptor, protein	A tale of two TRAPs: LAT and LAB in the regulation of lymphocyte development, activation, and autoimmunity
35	2014.5	4	intracapsular, silicone, silicone breast, silicone breast implant, breast implant, silicone implant, implant surface, fibrous capsule	Immunophenotypic characterization of human T cells after in vitro exposure to different silicone breast implant surfaces
36	2009.5	4	icodextrin, adhesion formation, peritoneal adhesion, parietal adhesion, peritoneal tissue response, ccl1, adhesion, atopic skin inflammation	Chronological evaluation of inflammatory mediators during peritoneal adhesion formation using a rat model
37	1988	3	leukocyte molecule, bdv, border disease, border disease virus, sheep leukocyte, sheep leukocyte molecule, efferent lymphocyte, t19	Cell phenotypes in the efferent lymph of sheep persistently infected with Border disease virus
38	1996	3	cfrs, htlv, tsp/ham, oral keratinocytes, tsp/ham patient, paraparesis/htlv, spastic paraparesis/htlv, tropical spastic paraparesis/htlv	Human T-Lymphotropic Virus (HTLV) Type I in vivo Integration in Oral Keratinocytes

Information on 38 clusters was obtained from the citation network analysis of papers published up until the end of 2020. The median publication year of constituent papers, characteristic keywords based on the term frequency-inverse cluster frequency (TF-ICF, see Methods), and the titles of hub papers with the highest number of citations in each cluster are listed

$$TFICF={tf}_{i,j}\cdot {icf}_{i}={tf}_{i,j}\cdot \mathrm{log}(N/{cf}_{i})$$

where N is the total number of sentences. Based on the keywords with high TF-ICF of each cluster, we can infer the topic of each cluster.

To confirm the trends in the research field, the mean and median year of publication of papers in each cluster were extracted, as well as information on journals, authors, and affiliated institutions.

After clustering the network, visualization was converted to intuitively infer relationships among these clusters. We use a large graph layout (LGL), which is based on a force-direct layout algorithm [29, 30]. This layout can display the largest connected component of the network to generate coordinates for nodes in two dimensions. We visualized a citation network by expressing inter-cluster links with the same color (Step 4 in Fig. 1). However, the position of the clusters and the distance between clusters do not indicate an approximation of the content. An overview of the diagram is presented in Fig. 1.

## Results

### Results of Citation Network Analysis

We analyzed a citation network of papers obtained from PubMed, and 38 clusters were formed. Table 2 shows the information on these clusters. The topic of each cluster can be inferred based on the characteristic keywords with high TF-ICF of each cluster, and the title and abstract of several papers that are most cited within each cluster. Summary of top 10 clusters, which contain 80% of the papers in all clusters, were suggested based on Table 2 as follows; Cluster 1 is assumed to be a group of papers on regulatory T cells, as the top keywords include “tregs” and “foxp3.” Cluster 2 includes “tumors” and “immunotherapy” in the top keywords, suggesting that it consists of papers related to cancer immunity. These two clusters have the most recent average year of publication, suggesting that they are topics that have been extensively discussed in recent times. Cluster 3 is assumed to be a group of papers on the immune response to HIV and SIV infection because the top keywords include “HIV”, “virus”, and “infection.” Cluster 4 is assumed to be a group of papers on immunosuppression because the top keywords include “suppressor” and “suppressor cells”. The average publication year of cluster 4 (1984) is the earliest among the top 10 clusters, indicating that this topic has been discussed over a long period. Cluster 5 is associated with infection, particularly hepatitis virus and influenza, as its top keywords include “HCV”, “HBV” and “influenza.” Cluster 6 is presumed to be related to the role of dendritic cells in the immune response, because its top keywords include “dendritic” and “tumor”. Cluster 7 is considered to be related to immunotolerance because its top keyword is “tolerant.” “Ebv” and “cmv” in its top keywords of Cluster 8 indicate that it is a group of papers on viral infection, transplantation, and Tcell responses. Cluster 9 includes “tuberculosis” and “mycobacterium” in the top keywords, suggesting that it is a group of papers related to bacterial infection and immunity. Cluster 10 might be associated with the role of helper T cells in the immune response, as the top keywords include “tfh” and “tcr.” Table 2 also shows the variation in the median publication year of the papers in each cluster: 1984 for Cluster 4, 1997 for Cluster 7, and 2008–2012 for others. The distribution of publication years over a long period of time is supposed to represent a characteristic of immunology field that research progress requires accumulation of a lot of research.

We selected the six key articles shown in Table 1 as studies that contributed to the research and development (R&D) of immune checkpoint inhibitors. By examining the clusters which contain these articles, we assessed whether clusters represent the contents of these articles: article A [24] on the discovery of PD-1, article B [23] on the function of CTLA-4, article C [22] that showed antitumor activity of anti-CTLA-4 antibody in mice, article D [21] on the involvement of PD-1 in autoimmunity; article E [20] on PD-1 and immunosuppression, and article F [19] on the antitumor effect of PD-L1 inhibitors in mice. Articles A, D, and E, all of which are related to immunotolerance were included in Cluster 5, which is associated with immune checkpoints in viral infection. The hub paper [31] of the cluster is on the functional recovery of CD8 + T cells by PD-1/PD-L1, with possible involvement in chronic viral infection. All of the key articles A, D, and E were included probably because all are related to immunotolerance. Subclusters obtained by reanalysis of Cluster 5 also shows the cluster contains papers on the function of immune checkpoints in restoring the function of T cells (Supplement 3). Key articles C and F were included in cluster 2, which is related to cancer therapy. These results suggested that the papers with similar content are classified in the same cluster. Article B was excluded from the analysis probably because CTLA-4 did not receive much attention until the anti-tumor effects of anti-CTLA-4 antibodies were demonstrated.

It is suggested that Cluster 2 might be related to cancer immunity.

### Tracking the Time Series of Key Articles

**Fig. 2:**
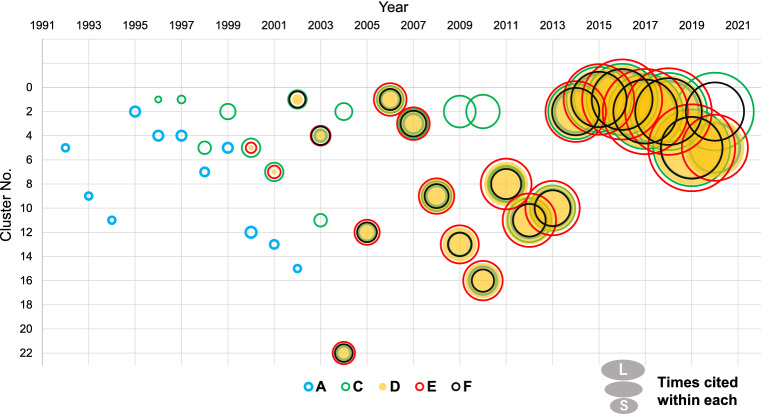
Tracking clusters containing key articles. Papers obtained from PubMed published up to the indicated year were analyzed. The cluster numbers that contained the five key articles shown in Table 1 were plotted, and the size of the circles represents the approximate number of citations in the cluster for each paper. The table of clusters containing each key article and its citation within the cluster is shown in Supplement 1

In order to validate the method by confirming whether it can visualize the research progress and demonstrate the overall research landscape of the immune field, we assessed the transition of research on immune checkpoint inhibitors by analyzing papers published up to each year and identified the cluster containing the key articles in Table 1 as well as the number of citations within the cluster. As shown in Fig. 2, articles A and C were initially classified into different clusters and had a small number of citations. After the publication of article D in 1999 and article E in 2000, articles C, D, and E were contained in the same cluster. In 2002, when article F was published, and later, all key articles were found in the same cluster until 2019, except for article A in 2002 and article C in 2003, 2004, 2009, and 2010. The number of citations of key articles in each cluster increased, indicating that they received more attention. From 2014 to 2019, all of them were detected in Clusters 1 and 2, and the number of citations continued to increase.

In this method, clusters are constructed based on papers with related citations; therefore, papers with several common citations, that is, papers that are likely to have similar content, are placed in the same cluster. The key articles in Table 1 were detected in either of two clusters according to the content of each paper indicates that the papers with similar content into the relevant clusters. It was also observed that the number of citations accumulated up to the analysis year indicating that interest in the research of immune checkpoint inhibitor increased annually. Lowering the cluster ranking, which represents the ranking of the number of papers in the cluster, was often observed (2007, 2008, 2009, 2010, 2012, and 2017 in Fig. 2). This is because the papers on a particular topic among related studies formed a new independent cluster, and we observed that the number of papers in clusters increased and the cluster numbers rose afterward (2013, 2014 in Fig. 2).

### Recent Research Trends in T Lymphocyte Immunology

To detect the latest research trends in T lymphocyte immunology in this area, we reanalyzed ‘young’ clusters, which contains more papers published recently. The cluster often includes papers with broadly related topics, and sub-clustering may reveal individual topics; therefore, we reanalyzed clusters 1 to 5, which are the top five clusters with the most papers included as well as clusters 15, 16, 17, and 19, which were the top five in terms of the percentage of papers published in the last 3 or 5 years among the papers in each cluster (Table 3). Research trends were analyzed over 3 and 5 years because based on the results of the analyses described above, the median year of publication of the papers included in the cluster was over 10 years ago (Table 2), it is supposed to be necessary to analyze trends over several years to capture the progress of research at a certain level. In addition, it may take up to a year from the date of publication before a paper is included in PubMed. Therefore, considering a short period of one to 2 years could lead to overlooking new research trends. We examined subclusters formed by the re-analysis with more than 100 papers and median publication year of 2016 or later (Table 4), since clusters with a small number of papers are considered to have relatively low research activity. Cluster 5 is related to immune tolerance to viral infections, and its subclusters 5–14 are related to TIGIT and CD155, which are considered to be the third molecules [32] for immune checkpoints following PD-1/PDL-1 and CTLA4/CD80/86, whose inhibitors have already been used in pharmaceuticals. Cluster 15 is related to immune responses to viral infections, and its subclusters 15–1 and 15–4 had a median publication year of 2020 for the constituent papers. Sub-cluster 15–1 was inferred to be related to T cell immunity against SARS-CoV2 infection and COVID-19. Sub-cluster 15–4 is likely to be related to the function of monocyte CD300e [33] and cancer ARID1A genes [34], which have recently attracted much attention. Cluster 16 is related to cellular immune responses to infection, and its sub-cluster 16–1 is presumably related to the function of tissue resident memory T cells and their application in vaccines and therapy. Cluster 19 is presumed to be related to the therapeutic application of cancer immunity, and its sub-cluster 19–1 includes papers on iRGD [35], an RGD peptide derivative that is expected to promote anticancer drug uptake by tumor cells. All subclusters obtained by reanalysis is demonstrated in Supplement 3.

**Table 3:** The six clusters with top 5% of papers in either the last 3 or 5 years

Cluster #	The number of papers	Median publication year	The number of papers in last 5 years	The number of papers in last 3 years	Published in last 5 years/All years	Published in last 3 years/All years
1	12,927	2012	3074	1423	0.238	0.110
2	12,911	2012	4075	2247	0.316	0.174
15	1222	2013	513	381	0.420	0.312
16	1205	2010	322	170	0.267	0.141
17	1177	2011	272	137	0.231	0.116
19	681	2016	343	312	0.504	0.458

As an indicator for clusters with more new papers, the ratio of papers published in the last 3 or 5 years versus all papers in each cluster was calculated, and the top five clusters in either category are shown. Clusters 1 and 17 were included in either 3 or 5 years, so a total of six clusters were targeted. The top five in either category are underlined

**Table 4:** Clusters with candidate new topics for horizon scanning

Cluster name	Median publication year	The number of papers	Top keywords	The title of hub papers
Cluster5	2009	7098	virus, hcv, infection, hepatitis, mouse, hbv, viral	Restoring function in exhausted CD8 T cells during chronic viral infection
Sub5-14	2016	157	tigit, cd155, cd226, dnam, cd96, cd112, nectin	The surface protein TIGIT suppresses T cell activation by promoting the generation of mature immunoregulatory dendritic cells
Cluster15	2013	1222	cell, response, immune, virus, infection, mouse, vaccine	Studies on production of biologically active substance which inhibits the intracellular multiplication of Toxoplasma within mouse macrophages
Sub15-1	2020	252	sars, cov, covid, sars cov, coronavirus, severe acute, respiratory, severe acute respiratory syndrome	T cell-mediated immune response to respiratory coronaviruses
Sub15-4	2020	134	tolvaptan, cd300e, arid1a, ataa, nbbs, flow immunotyping, sars	Studies on production of biologically active substance which inhibits the intracellular multiplication of Toxoplasma within mouse macrophages
Cluster16	2010	1205	cell, immune, response, mouse, infection, specific, lymphocyte	Memory T cells in nonlymphoid tissue that provide enhanced local immunity during infection with herpes simplex virus
Sub16-1	2017	235	resident memory, tissue resident memory, memory, trm, tissue resident, resident, trm cell	Memory T cells in nonlymphoid tissue that provide enhanced local immunity during infection with herpes simplex virus
Cluster19	2016	681	cell, immune, response, lymphocyte, tumor, patient, mouse	Uncoupling the proinflammatory from the immunosuppressive properties of tumor necrosis factor (TNF) at the p55 TNF receptor level: implications for pathogenesis and therapy of autoimmune demyelination
Sub19-1	2020	344	covid, tumor, cancer, irgd, pttg1, sars, mouse	Uncoupling the proinflammatory from the immunosuppressive properties of tumor necrosis factor (TNF) at the p55 TNF receptor level: implications for pathogenesis and therapy of autoimmune demyelination

The information on subclusters obtained from reanalysis of the top 10 clusters is shown in Table 3, with a median publication year of 2016 or later and with more than 100 papers

## Discussion

In this study, we applied citation network analysis and citation network analysis of bibliographic information in the field of immunology, which takes a rather long period to progress research due to the complexity of in vivo molecular interactions. For the immune checkpoints, CTLA-4 and PD-1 were identified in 1987 [36] and 1992 [24], respectively. It took more than 20 years for the approval of their inhibitors as pharmaceuticals in 2011 and 2014.

We were able to grasp the academic landscape of the field of immunology thorough the analysis as shown in Table 2 and Supplement 2. We then examined whether the development history of immune checkpoint inhibitors, which were developed as anti-cancer drugs with novel mechanisms of action, could be reproduced by this method. The following is a summary of the development history of immune checkpoint inhibitors and the relevant key articles: CTLA-4 was identified as an immunoglobulin superfamily molecule in 1987 [36] and PD-1 as an apoptosis-related immunoglobulin superfamily molecule by in 1992 [24], and research on their functions has continued. In 1995 [23], CTLA-4 was reported to be associated with T cells, and in 2000 [20], PD-1 was reported to be involved in autoimmune diseases.

Inhibitory antibodies against CTLA-4 and PD-1 were first reported to have anti-tumor effects in preclinical studies in 1996 [22] and 2002 [19], respectively. Clinical trials for ipilimumab, an anti-CTLA-4 antibody, were first started in the US in 2001 [37] and it was first approved in the US in 2011. Clinical trials for nivolumab, an anti-PD-1 antibody, were first started in the US in 2006 [38] and was first approved in Japan in 2014 [39]. Clinical trials for pembrolizumab, another anti-PD-1 antibody, were first conducted in the US in 2010 [40] and it was first approved in the US in 2014 [41]. In this study, we did not focus on anti-PD-L1 antibodies, which were developed later than anti-PD-1 antibodies because our purpose was to understand early research trends before the establishment of anti-cancer drugs based on the inhibition of immune checkpoints.

The results of our analysis during this period are shown in Fig. 2. From this figure, it can be seen that the cluster containing most of the key articles fluctuated significantly from 2002 to 2010, (2002, 1, 2003, 4, 2004, 22, 2005, 12, 2006, 1, 2007, 3, 2008, 9, 2009, 13, and 2010, 16 in Fig. 2). Such fluctuations may be accompanied by a decrease in the number of constituent papers and an increase in the citation number of key articles in the cluster when the number of papers on a particular topic in the cluster increases and, forming a new, independent cluster (2007, 2008, 2009, 2010, 2012, 2017, and 2020 in Fig. 2) (Fig. 2 2013, 2014). We confirmed that the fluctuations in Fig. 2 were caused due to the aforementioned reason by examining specific keywords and papers with a high number of citations in each cluster. Thus, the fluctuations in cluster number with increasing citation suggest a rapid progress of the topic, and the results shown in Fig. 2 suggest an increase in research activity several years prior to the commencement of the clinical trials of nivolumab and pembrolizumab in 2006 [38] and 2010 [40], respectively.

Figure 2 shows no significant changes for the number of citations of key article A on PD-1 prior to the clinical trial of ipilimumab, an ant-CTLA-4 antibody in 2000 [20]. One of the possible reasons is that the idea of using immune checkpoint inhibitors for cancer treatment was rather dubious at that time, because few research reports supported this idea. Significant activation of relevant research was not observed even after the clinical trial, since the efficacy of ipilimumab alone was not great enough to attract attention [42]. Tremendous research efforts over a long period were also required until the establishment of the concept of immune checkpoints [43], which is common to both CTLA-4 and PD-1. These results suggest that the analysis could reproduce the research progress of immune checkpoints and that the potential for product development could have been predicted several years before their clinical trials.

Next, we investigated recent research trends in this field (Table 4). Owing to the huge amount of funding for COVID-19-related research since early 2020, some of the ‘young’ subclusters were related to immune responses to coronavirus infection and COVID-19: sub15-1 (median publication year of constituent papers is 2020), sub15-7 (2018), which is excluded in Table 4 because it contains less than 100 papers, and sub19-1 (2020). Progress in research has been observed not only in the immunological response to SARS-CoV2 infection, but also in peripheral fields, including iRGD [35], an RGD peptide derivative that is expected to promote selective uptake of antitumor or antiviral drugs into tumor or infected cells (sub19-1). Research on immune checkpoint inhibitors seems to be still active, as suggested by sub5-14 (2016) related to TIGIT [32], another immune checkpoint molecule following CTLA-4 and PD-1, which is in clinical development. It is also suggested that monocyte CD300e [33] and cancer ARID1A gene [34] in sub15-4 (2020) may also be candidate topics that continue to follow. Monocyte CD300e [33] is a leukocyte mono-immunoglobulin-like receptor that recognizes lipids and is involved in allergy or inflammation. The ARID1A gene mutation is detected in some types of cancer in the ovary, stomach, or bile tract and is of interest as a target for anti-cancer drug development [34]. The function of resident memory T cells [44] and their application to vaccines and therapeutics [45] is also a possible new topic found in sub16-1 (2017). Recently, interest in the relationship between immune checkpoint molecules and predictive tumor biomarkers has recently attracted attention, and the accumulation of TILs in the tumor parenchyma [46] was observed in Cluster2, suggesting a candidate topic to follow up in the future. It is conceivable that these candidate topics should continue to be followed by periodical analysis, every several months, for example, because they might be applied to new drugs with novel mechanisms of action.

Thus, this study shows that citation network analysis and text mining using the methods of Sakata et al*.* [16] can be used to understand new research trends not only in fundamental technologies applicable to various fields such as nanocarbon and AI [18, 47], but also in biological fields elucidated over a long period, such as immunology.

This study is the first to show that this method can be used appropriately as a tool for horizon scanning in the medical field. Because it is difficult to extract a limited number of novel topics that may affect pharmaceutical regulations from a vast amount of information on a human basis, it is reasonable and appropriate to use a computer-based method such as the method used in this study as a primary screening [47].

We assume regulators as the end users of this method, which offers a new tool for horizon scanning to extract new topics from a huge database leading to the development of guidance and revisions to pharmaceutical regulations. This method might be applied to other government activities, such as HTA, research funding, and the business field.

There may be limitations to our approach. One is the time delay until the publication of the research results, because most recently published papers without citation relationships cannot be included in this analysis method. However, we consider its impact on horizon scanning, targeting the stage before clinical development. Since it generally takes a few years from when the data have a high possibility of product development, non-clinical proof of concept, for example, to the start of clinical trials, we consider that our method can predict technologies that may lead to clinical development based on mid- to long-term research trends, even taking into account the time delay for publication. Another limitation is the necessity of the evaluation by experts in multiple aspects, such as expected medical positioning and patent information in order to achieve the purpose of horizon scanning, because our proposed method is based on the analysis of bibliographic information. In addition, we have not confirmed whether this method can be applied to all research domains. Hence, it may be necessary to consider an appropriate strategy for the utilization of this method in each field.

We also suggest that selection of a bibliographic database is critical. We extracted scientific papers for citation network analysis from PubMed, which consists of more than 30 million citations to biomedical papers from MEDLINE, life science journals, and online books [48].We also tried to analyze papers extracted from WoS, which consists of 161 million citations across 254 disciplines beyond science, but the obtained papers contained only one of the six key articles, suggesting that the papers that represent the research trends in this field were not extracted as described in METHODS. When we applied this analysis method to the artificial intelligence (AI) field to investigate the R&D of AI-equipped medical devices, we obtained more useful information from WoS than from PubMed [47]. In the study of three-dimensional cell layering using this method, WoS provided information more information on the base material for adhesive culture and bioprinting equipment, while PubMed provided more information on cell functions (*data not shown*). This suggests that the bibliographic database for analysis needs to be chosen according to the target field.

Other methods are being explored to predict the future research activity of a certain topic. For example, in addition to cluster analysis, budding prediction was proposed to predict the growth of identified high-profile papers in the fields of solar cells and nanocarbons [17, 18].

Most of the other horizon scanning activities in the health science field have been limited to HTA including IHSI activity focus on post-clinical and pre-marketing technologies. Our study is new in that it focuses on technologies that would be in clinical development in a relatively short period of time, with the aim of identifying novel technologies that could have regulatory implications at an early stage. Mechanical analysis using citation networks and text mining is used in the science map reported by the Japanese National Institute of Science and Technology Policy (NISTEP). However, they aim to obtain a bird's eye view of the entire scientific field by the top 1% of papers in terms of citations using "co-citation". Sakata’s method seems to be more suitable for our purpose since the method applies ‘direct citation’, which has been reported to be the most appropriate for obtaining leading-edge information on trends, to all papers extracted from the database [47].

Thus, this study demonstrates that the proposed method of horizon scanning targeting technologies prior to clinical development based on citation network analysis and text mining is unique.

## Conclusion

This study showed that the citation network analysis and text mining of scientific papers by the methods of Sakata et al*.* can objectively identify the emergence of new topics and their development through periodic analysis. We identified several candidate topics such as iRGD, ARID1A, and CD300e, for which tracking future research progress and potential applications to pharmaceuticals are recommended. The obtained results establish an efficient primary screening tool for horizon scanning procedure that enables regulators to prepare for new technologies, potentially benefiting patients through earlier access to the innovative products.

## Supplementary Information

Below is the link to the electronic supplementary material.

Supplementary file1 (PDF 187 KB)

Supplementary file2 (PDF 208 KB)

Supplementary file3 (PDF 300 KB)
