# Supplementary material for: Study on Horizon Scanning by Citation Network Analysis and Text Mining: A Focus on Drug Development Related to T Cell Immune Response
Source: Ther Innov Regul Sci. 2021 Nov 22;56(2):230–43. doi: 10.1007/s43441-021-00351-3 (PMC8608232; doi:10.1007/s43441-021-00351-3)
Supplement: Supplementary file 1 — Supplementary file1 (PDF 187 KB) [file 43441_2021_351_MOESM1_ESM.pdf]

Supplement 1

The number of citations and the cluster number where the key articles were included (in parentheses) are shown by year.

Article	1992	1993	1994	1995	1996	1997	1998	1999	2000	2001	2002	2003	2004	2005	2006	2007	2008	2009	2010	2011	2012	2013	2014	2015	2016	2017	2018	2019	2020
A	5 (3)	9 (3)	11 (3)	2 (5)	4 (5)	4 (5)	7 (4)	5 (5)	12 (6)	13 (4)	15 (3)	4 (7)	22 (9)	12 (13)	1 (5)	3 (17)	9 (18)	13 (23)	16 (32)	8 (44)	11 (51)	10 (56)	2 (69)	1 (97)	1 (124)	2 (135)	2 (139)	5 (142)	5 (95)
C					1 (4)	1 (3)	5 (7)	2 (10)	5 (14)	7 (14)	1 (14)	11 (7)	2 (12)	12 (15)	1 (28)	3 (32)	9 (34)	2 (39)	2 (43)	8 (50)	11 (72)	10 (77)	2 (113)	1 (156)	1 (187)	2 (205)	2 (218)	5 (216)	2 (222)
D									5 (1)	7 (1)	1 (4)	4 (8)	22 (10)	12 (14)	1 (27)	3 (29)	9 (37)	13 (40)	16 (44)	8 (68)	11 (81)	10 (77)	2 (97)	1 (128)	1 (166)	2 (191)	2 (182)	5 (194)	5 (122)
E									5 (5)	7 (7)	1 (11)	4 (16)	22 (20)	12 (25)	1 (41)	3 (41)	9 (48)	13 (55)	16 (59)	8 (96)	11 (106)	10 (110)	2 (136)	1 (183)	1 (235)	2 (265)	2 (274)	5 (276)	5 (161)
F											1 (11)	4 (14)	22 (13)	12 (16)	1 (18)	3 (26)	9 (23)	13 (22)	16 (20)	8 (35)	11 (41)	10 (49)	2 (84)	1 (114)	1 (137)	2 (151)	2 (164)	5 (141)	2 (126)
