# Supplementary material for: Study on Horizon Scanning by Citation Network Analysis and Text Mining: A Focus on Drug Development Related to T Cell Immune Response
Source: Ther Innov Regul Sci. 2021 Nov 22;56(2):230–43. doi: 10.1007/s43441-021-00351-3 (PMC8608232; doi:10.1007/s43441-021-00351-3)
Supplement: Supplementary file 2 — Supplementary file2 (PDF 208 KB) [file 43441_2021_351_MOESM2_ESM.pdf]

Supplement 2 The top five clusters in the ratio of papers published in the last three or five years versus all papers in each cluster

As an indicator for clusters with more new papers, the ratio of papers published in the last three or five years versus all papers in each cluster was calculated, and the top five clusters in either category are underlined. Clusters with fewer than 100 papers are not included because it cannot be expected that reliable information will be obtained by subsequent sub-clustering.

Cluster #.	The number of papers	Median publication year	The number of papers in last 5 years	The number of papers in last 3 years	Published in last 5 years /All years	Published in last 3 years /All years
1	12927	2012	3074	1423	0.238	0.110
2	12911	2012	4075	2247	0.316	0.174
3	8452	2007	1139	522	0.135	0.062
4	7842	1984	665	536	0.085	0.068
5	7098	2009	1376	695	0.194	0.098
6	5859	2008	839	373	0.143	0.064
7	5804	1997	284	144	0.049	0.025
8	4901	2008	838	394	0.171	0.080
9	4019	2008	623	304	0.155	0.076
10	3289	2010	729	358	0.222	0.109
11	2800	2006	371	193	0.133	0.069
12	2191	2011	425	196	0.194	0.089
13	2050	2007	353	197	0.172	0.096
14	1363	2008	205	87	0.150	0.064
15	1222	2013	513	381	0.420	0.312
16	1205	2010	322	170	0.267	0.141
17	1177	2011	272	137	0.231	0.116
18	1083	2008	231	83	0.213	0.077
19	681	2016	343	312	0.504	0.458
20	652	2006	67	32	0.103	0.049
21	598	2005	33	11	0.055	0.018
22	571	2004	82	31	0.144	0.054
23	456	2005	32	10	0.070	0.022
24	384	2003	44	18	0.115	0.047
25	372	2005	33	14	0.089	0.038
26	256	2007	40	20	0.156	0.078
27	207	2009	32	16	0.155	0.077
