# Supplementary material for: Study on Horizon Scanning by Citation Network Analysis and Text Mining: A Focus on Drug Development Related to T Cell Immune Response
Source: Ther Innov Regul Sci. 2021 Nov 22;56(2):230–43. doi: 10.1007/s43441-021-00351-3 (PMC8608232; doi:10.1007/s43441-021-00351-3)
Supplement: Supplementary file 3 — Supplementary file3 (PDF 300 KB) [file 43441_2021_351_MOESM3_ESM.pdf]

Supplement 3 Details of clusters of new technologies

The details of the six clusters in Table 3 are shown. These clusters were top 5 clusters with the highest percentage of papers in the last three or five years. The subclusters are listed with more than 100 papers.

Cluster name		Median publication year	The number of papers	Top keywords	The title of hub papers
Cluster1		2012	14538	cell, tumor, immune, cancer, response, patient	Myeloid-derived suppressor cells as regulators of the immune system
	Sub1-1	2011	1650	th17, tcdd, autoimmune, mouse, ahr, treg	The orphan nuclear receptor RORgammat directs the differentiation program of proinflammatory IL-17+ T helper cells
	Sub1-2	2012	1550	intestinal, microbiota, gut, mouse, treg, regulatory, mucosal, commensal	A functionally specialized population of mucosal CD103+ DCs induces Foxp3+ regulatory T cells via a TGF-beta and retinoic acid-dependent mechanism.
	Sub1-3	2014	1482	treg, foxp3, ipex, tregs, regulatory, treg cell, reg, transcription	Control of regulatory T cell development by the transcription factor Foxp3
	Sub1-4	2010	1176	treg, helminth, infection, regulatory, tregs, mouse, parasite, filarial	CD4+CD25+ regulatory T cells control Leishmania major persistence and immunity
	Sub1-5	2006	1097	regulatory, treg, tregs, tolerance, cd25, cd4, girt, autoimmune	Immunologic self-tolerance maintained by activated T cells expressing IL-2 receptor alpha-chains (CD25). Breakdown of a single mechanism of self-tolerance causes various autoimmune diseases
	Sub1-6	2012	1045	fviii, treg, tregs, regulatory, tolerance, gvhd, graft, transplantation	Tregs prevent GVHD and promote immune reconstitution in HLA-haploidentical transplantation
	Sub1-7	2013	819	leptin, adipose tissue, adipose, mtor, metabolic metabolism, ogf, obesity	Lean, but not obese, fat is enriched for a unique population of regulatory T cells that affect metabolic parameters
	Sub1-8	2012	617	t1d, treg, diabetes, autoimmune, regulatory, mouse, sle, tregs	Interleukin-2 at the crossroads of effector responses, tolerance, and immunotherapy.
	Sub1-9	2013	592	th9 cell, th9, tl1a, notch, st2, tslp, notch signaling, mouse	The development and in vivo function of T helper 9 cells
	Sub1-10	2012	581	treg, ctla, tregs, malt1, regulatory, lrba, mouse, pah	CTLA-4 control over Foxp3+ regulatory T cell function
	Sub1-11	2009	441	regulatory, treg, tregs, cd8, tnfr2, cd8+, autoimmune, cd4	CD8(+) Tregs in autoimmunity: learning "self"-control from experience
	Sub1-12	2014	427	mir, mirnas, roquin, microrna, mirna, micrornas, drak2, itch	Foxp3-dependent microRNA155 confers competitive fitness to regulatory T cells by targeting SOCS1 protein
	Sub1-13	2010	338	allergen, allergen specific, sit, allergic, slit, immunotherapy, peanut, oit	Role of interleukin 10 in specific immunotherapy
	Sub1-14	2012	273	b10 cell, regulatory, bregs lit, b10, mouse, bcdm, pf4	A regulatory B cell subset with a unique CD1dhiCD5+ phenotype controls T cell-dependent inflammatory responses
	Sub1-15	2011	264	vitamin, vdr, dihydroxyvitamin, psc, primary sclerosing, primary sclerosing cholangitis, calcitriol, cholangitis	Vitamin D Actions on CD4(+) T Cells in Autoimmune Disease
	Sub1-16	2010	200	garp, tgf, klf10, treg, tregs, smad3, smad4, regulatory	TGF-beta1 maintains suppressor function and Foxp3 expression in CD4+CD25+ regulatory T cells

	Sub1-17	2013	142	stroke, ich, brain, intracerebral hemorrhage, cerebral, mcao, infarct, stroke patient	Regulatory T cells are key cerebroprotective immunomodulators in acute experimental stroke
	Sub1-18	2011	111	itp, itp patient, gpiib, gpiib iiia, platelet, immune thrombocytopenia, iiia, thrombocytopenia	Defective circulating CD25 regulatory T cells in patients with chronic immune thrombocytopenic purpura
Cluster2		2012	12911	tumor, cancer, immunotherapy, therapy, melanoma, vaccine, peptide, patient	Improved survival with ipilimumab in patients with metastatic melanoma
	Sub2-1	2017	2569	tumor, ipilimumab, cancer, checkpoint, melanoma, immunotherapy, ctla, therapy	Improved survival with ipilimumab in patients with metastatic melanoma
	Sub2-2	2009	2143	tumor, cancer, treg, tregs, immunotherapy, regulatory, vaccine, patient	Specific recruitment of regulatory T cells in ovarian carcinoma fosters immune privilege and predicts reduced survival
	Sub2-3	2012	1266	mdscs, mdsc, tumor, derived suppressor, derived suppressor cell, myeloid derived suppressor, myeloid derived suppressor cell, myeloid derived	Myeloid-derived suppressor cells as regulators of the immune system
	Sub2-4	2014	1187	tumor, cancer, colorectal, colorectal cancer, survival, tils, infiltrating, crc	Type, density, and location of immune cells within human colorectal tumors predict clinical outcome
	Sub2-5	2015	1014	car, tumor, chimeric antigen receptor, therapy, chimeric antigen, immunotherapy, cancer, cell therapy	Chimeric antigen receptor-modified T cells in chronic lymphoid leukemia
	Sub2-6	2007	1012	tumor, peptide, pdt, melanoma, vaccine, cancer, hla, mage	Simultaneous humoral and cellular immune response against cancer-testis antigen NY-ESO-1: definition of human histocompatibility leukocyte antigen (HLA)-A2-binding peptide epitopes
	Sub2-7	2008	720	tumor, vaccine, hpv, cancer, dna, peptide, mcpyv, vaccination	Antigen-specific immunotherapy of cervical and ovarian cancer
	Sub2-8	2009	461	cd137, tumor, ox40, anti cd137, anti cd137 mab, ox40l, cd137 mab, cd137l	4-1BB Agonists: Multi-Potent Potentiators of Tumor Immunity
	Sub2-9	2012	410	tumor, rhamm, sorafenib, sunitinib, hcc, gpc3, cea, therapy	Production of vascular endothelial growth factor by human tumors inhibits the functional maturation of dendritic cells
	Sub2-10	2015	313	pancreatic cancer, pdac, tumor, pancreatic, pda, ductal adenocarcinoma, pancreatic ductal adenocarcinoma, cancer	CD40 agonists alter tumor stroma and show efficacy against pancreatic carcinoma in mice and humans
	Sub2-11	2015	246	tumor, immunogenic cell death, icd, immunogenic cell, autophagy, cancer, death, l19	Consensus guidelines for the detection of immunogenic cell death
	Sub2-12	2010	203	nkg2d, tumor, smic, mica, nkg2d ligand, muc16, cancer, letal	The Paradoxical Role of NKG2D in Cancer Immunity
	Sub2-13	2011	200	tumor, dci, her2, cetuximab, cancer, ly6k, peptide, ttk	The therapeutic effect of anti-HER2/neu antibody depends on both innate and adaptive immunity
	Sub2-14	2009	198	oncolytic, tumor, g207, ctla, sctla, reovirus, oncolytic virus, vsv	Oncolytic immunovirotherapy for melanoma using vesicular stomatitis virus.
	Sub2-15	2014	192	tumor, csc, hifu, glioma, cscs, rfa, cancer, sox2	Antigen-specific T-cell response from dendritic cell vaccination using cancer stem-like cell-associated antigens
	Sub2-16	2013	175	sepsis, septic, septic shock, shock patient, septic shock patient, clp, sepsis induced, patient with sepsis	Sepsis-induced apoptosis causes progressive profound depletion of B and CD4+ T lymphocytes in humans

	Sub2-17	2011	168	cll, cll cell, lenalidomide, Ibrutinib, chronic lymphocytic, ror1, chronic lymphocytic leukemia, lymphocytic leukemia	Chronic lymphocytic leukemia T cells show impaired immunological synapse formation that can be reversed with an immunomodulating drug.
	Sub2-18	2006	163	tumor, tdln cell, r24, tdln, mpe, ifl, carbonica, gangliosides	Tumor-shed PGE(2) impairs IL2Rgammac-signaling to inhibit CD4 T cell survival: regulation by theaflavins
	Sub2-19	2013	148	adenosine, a2ar, tumor, cd73, a2a, adenosine receptor, cd39 adenosinergic	A2A adenosine receptor protects tumors from antitumor T cells
Cluster5		2009	7098	virus, hcv, infection, hepatitis, mouse, hbv, viral	Restoring function in exhausted CD8 T cells during chronic viral infection
	Sub5-1	2013	1017	tumor, ctla, death, checkpoint, programmed, cd200, hiv, lag	Restoring function in exhausted CD8 T cells during chronic viral infection
	Sub5-2	2004	743	lcmv, virus, infection, mouse, viral, choriomeningitis, lymphocytic choriomeningitis, choriomeningitis virus	Viral immune evasion due to persistence of activated T cells without effector function
	Sub5-3	2009	725	memory, cd8, effector, infection, cd8+, memory cell, mouse, cell response	Inflammation directs memory precursor and short-lived effector CD8(+) T cell fates via the graded expression of T-bet transcription factor
	Sub5-4	2010	712	influenza, virus, influenza virus, vaccine, infection, h1n1, heterosubtypic, h5n1	Cellular immune correlates of protection against symptomatic pandemic influenza
	Sub5-5	2008	700	hcv, hcv specific, hepatitis, hcv infection, virus, ns3, infection, hcv core	Analysis of successful immune responses in persons infected with hepatitis C virus
	Sub5-6	2009	533	hbv, hepatitis, hbsag, hbv specific, hbcag, hbeag, hbv infection, woodchuck	Hepatitis B virus immunopathogenesis
	Sub5-7	2007	373	tcr, lcmv, virus, peptide, memory, repertoire, heterologous immunity, infection	Heterologous immunity between viruses
	Sub5-8	2004	366	drone, tolerance, mouse, tcr, srih, antigen, peptide, nfat1	Class I-restricted cross-presentation of exogenous self-antigens leads to deletion of autoreactive CD8(+) T cells
	Sub5-9	2013	295	tim, galectin, sema4a, mucin domain, anti tim, cell immunoglobulin, mucin, tim3	Interaction of Tim-3 and Tim-3 ligand regulates T helper type 1 responses and induction of peripheral tolerance
	Sub5-10	2009	282	liver, lsec, aih, cyp2d6, lsecs, hepatitis, lsectin, hepatic	Modulation of liver tolerance by conventional and nonconventional antigen-presenting cells and regulatory immune cells
	Sub5-11	2003	230	bdv, mhv, jhm, borna, borna disease, jhmv, virus, borna disease virus	The pathogenesis of murine coronavirus infection of the central nervous system
	Sub5-12	2008	222	allograft, rejection, memory, transplant, recipient, transplantation, graft, donor reactive	Heterologous immunity provides a potent barrier to transplantation tolerance
	Sub5-13	2009	199	vacv, smallpox, virus, dryvax, ectv, vaccinia, smallpox vaccine, vaccinia virus,	Identification of poxvirus CD8+ T cell determinants to enable rational design and characterization of smallpox vaccines.
	Sub5-14	2016	157	tigit, cd155, cd226, dnam, cd96, cd112, nectin	The surface protein TIGIT suppresses T cell activation by promoting the generation of mature immunoregulatory dendritic cells
	Sub5-15	2012	142	diffdcs, nkg2h, virus, infection, pmm2, pmm2 cdg, pegifnα, hbv	NK cells and their ability to modulate T cells during virus infections
	Sub5-16	2010	135	hvem, btla, entry mediator, herpesvirus entry, herpesvirus entry mediator,	B and T lymphocyte attenuator regulates T cell activation through interaction with herpesvirus entry mediator.

				light, tnfsf14, lymphocyte attenuator	
Cluster15		2013	1222	cell, response, immune, virus, infection, mouse ,vaccine	Studies on production of biologically active substance which inhibits the intracellular multiplication of Toxoplasma within mouse macrophages
	Sub15-1	2020	252	sars, cov, covid, sars cov, coronavirus, severe acute, respiratory, severe acute respiratory syndrome	T cell-mediated immune response to respiratory coronaviruses
	Sub15-2	2014	183	dengue, denv, zikv, dengue virus, zika, zikv infection, zika virus, flavivirus	A protective role for dengue virus-specific CD8+ T cells
	Sub15-3	2010	175	complement, daf, c3a, c3ar, c5a, cr2, c5ar, cr1	Locally produced C5a binds to T cell-expressed C5aR to enhance effector T-cell expansion by limiting antigen-induced apoptosis
	Sub15-4	2020	134	tolvaptan, cd300e, arid1a, ataa, nbbs , flow immunotyping ,sars	Studies on production of biologically active substance which inhibits the intracellular multiplication of Toxoplasma within mouse macrophages
	Sub15-5	2011	131	wnv, west nile virus, west nile, nile, nile virus, flavivirus, virus, west	Role of CD8+ T cells in control of West Nile virus infection
Cluster16		2010	1205	cell, immune, response, mouse, infection, specific, lymphocyte	Memory T cells in nonlymphoid tissue that provide enhanced local immunity during infection with herpes simplex virus
	Sub16-1	2017	235	resident memory, tissue resident memory, memory, trm, tissue resident, resident, trm cell	Memory T cells in nonlymphoid tissue that provide enhanced local immunity during infection with herpes simplex virus
	Sub16-2	2007	191	chlamydia, trachomatis, chlamydial, chlamydia trachomatis, muridarum, mopn, genital, momp	Immunity to murine Chlamydia trachomatis genital tract reinfection involves B cells and CD4(+) T cells but not CD8(+) T cells
	Sub16-3	2009	127	acd, contact, chs, contact dermatitis, dpcp, allergic contact dermatitis, allergic contact, skin	IL-17 and IFN-gamma mediate the elicitation of contact hypersensitivity responses by different mechanisms and both are required for optimal responses
	Sub16-4	2008	111	smx, abacavir, drug, sulphamethoxazole, adrs, drug hypersensitivity, drug reaction, nevirapine	Idiosyncratic adverse drug reactions: current concepts
Cluster17		2011	1177	atherosclerosis, hypertension, apoe, atherosclerotic, mouse plaque, hypertensive, dahl	Role of the T cell in the genesis of angiotensin II induced hypertension and vascular dysfunction
	Sub17-1	2010	287	atherosclerosism, apoe, atherosclerotic, plaque, oxldl, aorta, atherogenesis, ldlr	The influence of innate and adaptive immune responses on atherosclerosis
	Sub17-2	2007	194	hypertension, dahl, salt sensitive, hypertensive, ang, angiotensin, shr, pressure elevation	Role of the T cell in the genesis of angiotensin II induced hypertension and vascular dysfunction
	Sub17-3	2009	102	ach, α7nachr, nicotine, alpha7, nachr, cgrp, nicotinic, cholinergic system	Acetylcholine-synthesizing T cells relay neural signals in a vagus nerve circuit

Cluster19		2016	681	cell, immune, response, lymphocyte, tumor, patient, mouse	Uncoupling the proinflammatory from the immunosuppressive properties of tumor necrosis factor (TNF) at the p55 TNF receptor level: implications for pathogenesis and therapy of autoimmune demyelination.
	Sub19-1	2020	344	covid, tumor, cancer, irgd, pttg1, sars, mouse	Uncoupling the proinflammatory from the immunosuppressive properties of tumor necrosis factor (TNF) at the p55 TNF receptor level: implications for pathogenesis and therapy of autoimmune demyelination.
